# Targeting B7-H3—A Novel Strategy for the Design of Anticancer Agents for Extracranial Pediatric Solid Tumors Treatment

**DOI:** 10.3390/molecules28083356

**Published:** 2023-04-11

**Authors:** Petar Rasic, Marija Jeremic, Rada Jeremic, Marija Dusanovic Pjevic, Milica Rasic, Slavisa M. Djuricic, Maja Milickovic, Miroslav Vukadin, Tanja Mijovic, Djordje Savic

**Affiliations:** 1 Department of Abdominal Surgery, Mother and Child Health Care Institute of Serbia “Dr. Vukan Cupic”, 11000 Belgrade, Serbia; majacvetkovicmilickovic@gmail.com (M.M.); miroslav_vukadin@yahoo.com (M.V.); tanja.sav@gmail.com (T.M.); djordje.savic@imd.org.rs (D.S.); 2 Institute of Medical and Clinical Biochemistry, Faculty of Medicine, University of Belgrade, 11000 Belgrade, Serbia; marija.jeremic@med.bg.ac.rs; 3 Institute of Medical Physiology “Richard Burian”, Faculty of Medicine, University of Belgrade, 11000 Belgrade, Serbia; rada.jeremic@med.bg.ac.rs; 4 Institute of Human Genetics, Faculty of Medicine, University of Belgrade, 11000 Belgrade, Serbia; marija.dusanovic-pjevic@med.bg.ac.rs (M.D.P.); milica.rasic@med.bg.ac.rs (M.R.); 5 Department of Clinical Pathology, Mother and Child Health Care Institute of Serbia “Dr. Vukan Cupic”, 11000 Belgrade, Serbia; slavisa.djuricic@med.unibl.org; 6 Faculty of Medicine, University of Banja Luka, 78000 Banja Luka, Bosnia and Herzegovina; 7 Faculty of Medicine, University of Belgrade, 11000 Belgrade, Serbia

**Keywords:** B7-H3, pediatric solid tumors, anticancer agents, targeted therapy, immunotherapy

## Abstract

Recent scientific data recognize the B7-H3 checkpoint molecule as a potential target for immunotherapy of pediatric solid tumors (PSTs). B7-H3 is highly expressed in extracranial PSTs such as neuroblastoma, rhabdomyosarcoma, nephroblastoma, osteosarcoma, and Ewing sarcoma, whereas its expression is absent or very low in normal tissues and organs. The influence of B7-H3 on the biological behavior of malignant solid neoplasms of childhood is expressed through different molecular mechanisms, including stimulation of immune evasion and tumor invasion, and cell-cycle disruption. It has been shown that B7-H3 knockdown decreased tumor cell proliferation and migration, suppressed tumor growth, and enhanced anti-tumor immune response in some pediatric solid cancers. Antibody-drug conjugates targeting B7-H3 exhibited profound anti-tumor effects against preclinical models of pediatric solid malignancies. Moreover, B7-H3-targeting chimeric antigen receptor (CAR)-T cells demonstrated significant in vivo activity against different xenograft models of neuroblastoma, Ewing sarcoma, and osteosarcoma. Finally, clinical studies demonstrated the potent anti-tumor activity of B7-H3-targeting antibody-radioimmunoconjugates in metastatic neuroblastoma. This review summarizes the established data from various PST-related studies, including in vitro, in vivo, and clinical research, and explains all the benefits and potential obstacles of targeting B7-H3 by novel immunotherapeutic agents designed to treat malignant extracranial solid tumors of childhood.

## 1. Introduction

Childhood cancer is defined as the group of cancer that arises between birth and nineteen years of age and involves nearly 400,000 new cases yearly [1]. Solid tumors represent half of all malignant neoplasms of childhood and comprise the central nervous system (CNS) and extracranial solid tumors. Neuroblastoma (NB), nephroblastoma (also known as Wilms tumor (WT)), and sarcomas, including rhabdomyosarcoma (RMS), osteosarcoma (OS), and Ewing sarcoma (ES), are the most common extracranial solid tumors in the pediatric population [2]. Poor survival of patients with high-grade, refractory, or metastatic pediatric solid tumors (PSTs) despite currently available combinations of surgery, cytotoxic chemotherapy, and radiation has led to an overflow of research seeking new therapeutic modalities [2,3,4,5,6,7].

The last decade brought advancements in the development of targeted immunotherapy significantly improving the prognosis of many adult solid tumors. Despite these results, immunotherapy for extracranial solid tumors of childhood remains in the early phase of development, and its clinical significance has yet to be examined. The principal reasons for the lower success of anticancer immunotherapy in children are the low tumor mutational burden and the limited number of actionable or targetable mutations in PSTs. Moreover, PSTs are characterized by a paucity of tumor-infiltrating lymphocytes (TILs) which makes them resistant to immunotherapeutic modalities such as immune checkpoint inhibitors. With the absence of TILs, the expression of programmed cell death protein 1 (PD-1), programmed cell death ligand-1 (PD-L1), and programmed cell death ligand-2 (PD-L2) is also low across PSTs and research has been conducted to find new checkpoint molecules that can be used as a target [3].

The results of many recent studies reveal that the B7 homolog 3 (B7-H3) checkpoint molecule seems to be an attractive target for immunotherapy in various adult cancers [8,9]. This molecule was also confirmed to be expressed in extracranial PSTs such as NB, RMS, WT, OS, and ES [4,10,11,12], as well as in childhood CNS tumors [13,14,15], whereas its expression is absent or very low in normal tissues and organs [16]. Consequently, different studies identified the potential of B7-H3 as a therapeutic target in both CNS [4,7,13] and non-CNS PSTs [4,6,17,18,19]. In this review, we summarize the established data from various pediatric cancer-related studies including in vitro, in vivo, and clinical research, and explain all the benefits and potential obstacles of targeting B7-H3 by novel immunotherapeutic agents designed to treat malignant extracranial solid tumors of childhood.

## 2. B7-H3

B7-H3 (also known as CD276) is a type I transmembrane glycoprotein discovered in 2001 as a homolog of the B7 family molecules [9]. It involves two extremely similar isoforms in humans, 2Ig-B7-H3 and 4Ig-B7-H3, determined by their extracellular domain, which comprises one or two identical pairs of immunoglobulin variable (IgV)-like and an immunoglobulin constant (IgC)-like domains. This molecule is encoded by a gene located in chromosome 15 and B7-H3 mRNA is found in most normal tissues [8,20]. However, the B7-H3 protein is relatively rarely expressed in physiological conditions, probably due to the activation of post-transcriptional regulatory mechanisms [8,21]. Several studies discovered that particular types of microRNA (miRNA) could suppress B7-H3 expression by targeting the B7-H3 3′-untranslated mRNA region (3′ UTR), which may explain the way of controlling the expression of this molecule [22,23,24]. B7-H3 is found to be constitutively expressed on amniotic fluid stem cells, osteoblasts, endothelial cells, and nonimmune resting fibroblasts. In addition, B7-H3 expression could be induced in T and B lymphocytes, natural killer (NK) cells, and antigen-presenting cells, such as dendritic cells and macrophages [21].

On the other hand, B7-H3 is widely expressed among cancer cells of various adult human malignancies including colorectal cancer, gastric cancer, esophageal cancer [9], pancreatic cancer [25], liver cancer [26], lung cancer [27], breast cancer [28], ovarian cancer [29], prostatic cancer [30], and renal cell carcinoma [31]. Although the expression of this molecule in PSTs is much less examined, the studies verify that B7-H3 is also overexpressed in CNS tumors [14] and extracranial solid tumors of childhood [4,10,11]. Furthermore, B7-H3 is proven to influence the biological behavior of many types of cancer through various immunological and nonimmunological molecular mechanisms. This molecule impacts the biological functions of immune system cells, including CD8^+^ T cells, CD4^+^ T cells, γδ T cells, CD45RO^+^ T cells, macrophages, and NK cells, and plays a pivotal role in regulating the innate and adaptive immune responses [9,32,33,34]. Although previously recognized as a costimulatory molecule that promotes T cell activation and interferon-gamma (IFN-γ) production [35], the majority of currently available data confirm that B7-H3 exerts coinhibitory effects on T cell responses, which may allow tumor cells to evade immune destruction [9,33,34,36]. Furthermore, by modulating intracellular signal transduction pathways, such as Janus kinase/signal transducer and activator of transcription (JAK/STAT), phosphatidylinositol 3-kinase/protein kinase B (also known as Akt)/mammalian target of rapamycin (PI3K/Akt/mTOR), extracellular signal-regulated kinase (ERK), and nuclear factor-Κb (NF-Κb), B7-H3 may influence cancer cell metabolism, and promote invasion, metastasis, and resistance to anticancer therapy [9,37,38,39,40,41]. Therefore, the presence of B7-H3 has been correlated with a worse prognosis in many solid malignant tumors [9,42]. Over the past few years, many attempts have been made to interrupt the B7-H3 functions in cancer cells and find an adequate B7-H3-related therapeutic modality that will provide better survival for patients with various malignancies [4,6,8,43,44,45,46].

## 3. Regulation of B7-H3 Expression in Extracranial PSTs

The B7-H3 molecule was shown to be widely expressed on extracranial PSTs [4,12,33,43,44]. While the B7-H3 transcript is ubiquitously expressed in a wide range of solid tumors as well as in normal tissues, the B7-H3 protein is primarily expressed only in tumor tissues [8].

Regulation of gene expression includes mechanisms that control the intensity and mode of gene activity in certain conditions and specific cells. Although regulation of gene expression at the pre-transcriptional and transcriptional levels has been the most explored [47], current studies are increasingly focused on the examination of post-transcriptional gene regulation [48,49]. Many molecules among which are numerous mRNA-binding proteins and miRNAs are included in different post-transcriptional regulatory mechanisms of gene expression such as alternative mRNA splicing, mRNA polyadenylation, mRNA stability, and protein translation [48,50].

MiRNAs are a major class of small noncoding RNA molecules, usually called “master regulators” of gene expression [51,52]. It has been projected that miRNAs regulate the expression of nearly 30% of the protein-coding genes [53]. In most cases, they recognize and bind to specific sequences at the 3′ UTR of their target mRNAs to induce translational repression and mRNA deadenylation and decapping [54]. In addition to numerous physiological functions, miRNAs have been linked to many diseases, among others, tumor formation [55,56]. Alterations in miRNA expression have been confirmed in different cancers [57,58]. Abnormal miRNA expression in cancer may be either detected as down- or upregulation, meaning various families of these molecules could act either as oncogenes or as tumor suppressors [53]. Forasmuch as the abovementioned roles of miRNAs as regulators of cancer formation and metastasis, different studies have investigated the relationship between miRNA and B7-H3, as a carcinogenesis-associated protein, primarily expressed only in tumor tissues [22,23,59,60]. It has been noticed that a specific member of the miRNA family, miR-29a, was vastly expressed in normal tissues, but downregulated in different solid tumors, including sarcomas, NB, and brain tumors. Additionally, B7-H3 protein expression correlated contrariwise with miR-29 levels in these tumors [22]. Results of different studies confirmed that miR-29 miRNAs downregulate B7-H3 expression by targeting B7-H3 3′UTR, which contains a miR-29 binding site [22,59,60]. Finally, Cheung et al. demonstrated the miR-29 downregulation in NB metastatic to the CNS compared with primary tumors. MiR-29 downregulation in metastatic NB was also associated with increased expression of several oncoproteins, including B7-H3, confirming once again the metastatic role and way of expression control of the B7-H3 molecule [61].

Other molecules that could act as transcriptional regulators, microRNA sponges, and protein templates, are circular RNAs (circRNAs) [62]. Acting as miRNA sponges, circRNAs could compete with endogenous RNAs in regulating post-transcriptional levels of gene expression. It was noticed that hsa_circ0021347 circRNA is downregulated in OS and in a strong converse relationship with B7-H3 expression in OS tissue [63].

Furthermore, the expression of B7-H3 was also related to some tumor-specific mutations [64]. Previous studies discovered a range of genetic alterations in pediatric cancer; some of which may be found in several types of PSTs (such as the *TP53* mutation), while others are tumor-specific [65]. The literature search reveals two studies that examined the association between B7-H3 expression and tumor-specific genetic mutations in extracranial PSTs, namely in RMS [64]. RMS is the most common pediatric soft tissue sarcoma comprising 5–8% of all childhood cancers. According to the latest World Health Organization-defined histological criteria, RMS is further subdivided into four subgroups, including embryonal, alveolar, spindle cell/sclerosing, and pleomorphic RMS, with the latter one only found in the adult population. Most alveolar RMS (ARMS) are characterized by the presence of translocations t(2;13)(q35;q14) or t(1;13)(p36;q14) [3]. These translocations create fusion genes that produce chimeric proteins containing the strong transcriptional transactivation domain from FOXO1 and the DNA-binding domains of PAX3 or PAX7 [66]. One group investigated the relationship between B7-H3 and PAX3-FOXO1 in ARMS. It is well known that expression of PAX3-FOXO1 contributes to poor prognosis in patients with ARMS, but the role of B7-H3 in this tumor was not previously elucidated. The authors showed that the knockdown of *PAX3-FOXO1* decreased the expression of B7-H3 in the ARMS cell line, indicating that PAX3-FOXO1 positively regulates B7-H3 expression. Additionally, they showed that expression of B7-H3 and PAX3-FOXO1 is associated with the upregulation of the pathways crucial for tumor cell migration [64]. The other study, performed by Lavoie et al., identified the expression of B7-H3 in 91.5% (122/132) of RMS tumor specimens. However, there was no statistically significant difference between PAX3/7-FOXO1 fusion-positive and -negative RMS in B7-H3 expression [33]. The above-mentioned opposite findings demand a further investigation regarding the relationship between genetic mutations in RMS and B7-H3 expression. This might lead to discovering the additional genetic factors that contribute to the regulation of B7-H3 expression.

## 4. B7-H3 and the Immunity in Extracranial PSTs

Although the basic B7-H3-related immunological molecular mechanisms are far less examined in childhood cancers than in adult ones [9,67,68,69,70], recent studies have begun to discover the influence of this molecule on immune evasion in PSTs [12,33,71].

The immune system plays a pivotal role in detecting and destroying transformed cells. Tumor cell elimination, as well as immune evasion, are determined by complex interactions between cancer cells and the tumor microenvironment (TME). The TME represents a complex and dynamic system consisting of tumor cells, endothelial cells, fibroblasts, and immune cells (including T and B lymphocytes, NK cells, monocytes and macrophages, myeloid-derived suppressor cells, and dendritic cells). Different subsets of TILs suppress or stimulate tumor growth and metastasis through direct interactions and the production of soluble molecules including growth factors, cytokines, and chemokines. Apart from the possible suppression of anti-tumor responses by some types of TILs, the immune system might also promote tumor development through a selection of less immunogenic, unstable variants [72].

Most currently available scientific data recognize the B7-H3 molecule as a suppressive factor in anti-tumor immunity [9,33,36]. Moreover, evaluation of the type, number, and location of TILs within the tumor has considerable prognostic value in various adult malignancies [73]. A negative correlation between cytotoxic and effector lymphocyte tumor infiltration and B7-H3 expression was proved in many adult tumors [74,75,76,77,78].

PSTs are generally considered immunologically “cold” because of their low immunogenicity, which can be attributed to a low mutational burden and consequently low neoepitope expression, as well as low expression of major histocompatibility complex class I (MHC-I). This low immunogenicity leads to a deficiency in lymphocyte tumor infiltration and an insufficient anti-tumor reactivity of the few TIL present [79].

Recent research proved that the additional factor contributing to the regulation of tumor lymphocyte infiltration in PSTs might be the expression of B7-H3 by cancer cells. The B7-H3 overexpression was associated with lower CD8^+^ T cell infiltration in adult tumors such as gastric, esophageal, prostate, and breast cancers [75,77,78,80]. It was also found that B7-H3 expression in RMS and OS was inversely correlated with the density of infiltrating CD8^+^ T lymphocytes [12,33]. Furthermore, Lavoie et al. found that B7-H3 knockout in RMS tumor cells increased T cell-mediated cytotoxicity [33]. Another group of authors examined the dynamics of B7-H3 expression regarding tumor progression in nude mice with OS. The expression and distribution of B7-H3 changed in the early, middle, and late stages of tumor formation. Reverse transcription polymerase chain reaction (RT-PCR) and Western blotting showed that B7-H3 expression was lower in the early and middle stages of tumor development, while B7-H3 mRNA and protein were overexpressed in the late stage. Additionally, they noticed that the number of CD3^+^ T cells statistically significantly decreased in the late phase of the disease [71]. These results prove that B7-H3 not only promotes OS progression but may be vital in suppressing immune surveillance in pediatric bone and soft tissue sarcoma [12,33,71].

Regulatory T lymphocytes (Tregs) are another subset of T cells that, as a component of the TME, can modulate anti-tumor immunity. In physiological conditions, these cells play an essential role in maintaining immune tolerance and controlling the inflammatory response. However, in the TME, Tregs may inhibit anti-tumor immunity by suppressing the function of immune effector cells through a variety of mechanisms [81]. Previous studies on adult patients showed a positive correlation between Treg infiltration and B7-H3 expression in esophageal carcinoma and non-small cell lung cancer [77,82]. Lavoie et al. observed similar findings, detecting that B7-H3-rich PAX3/7-FOXO1 fusion-positive RMS had a higher Treg infiltration, indicating the immunosuppressive role of B7-H3 in these tumors [33].

NK cells constitute 5–10% of the peripheral blood and take an important part in the anti-tumor immune response. They represent cytotoxic effectors able to recognize and destroy a range of different cancer stem cells and undifferentiated or poorly differentiated tumor cells as well as to recognize and lyse virus-infected cells. NK cells exert cytotoxic effects by releasing perforin and granzyme B which lead to necrosis or apoptotic cell death in susceptible targets. These cells also mediate direct and antibody-dependent cellular cytotoxicity (ADCC) against tumors, and they can also regulate the functions of other cells through the secretion of chemokines and cytokines [83]. Among cytotoxic lymphocytes, NK cells represent the most potent anti-tumor effectors and promising weapons against aggressive tumors such as NB. Having a low expression of MHC-I molecules, NBs are resistant to cytotoxic T-cell anti-tumor activity. Since NK cells are especially important in targeting and lysing MHC-I deficient cells, the modulation of their activity could be promising in the treatment of NB [84]. In the study by Castriconi et al., 4Ig-B7-H3 molecules expressed at the NB cell surface exerted a protective role from NK cell-mediated lysis, proving the immunosuppressive role of B7-H3. The authors also showed that this inhibitory effect could be reversed by masking the 4Ig-B7-H3 with monoclonal antibody (mAb) [34], which could potentially be used in anti-tumor targeted therapy. On the contrary, it was found that B7-H3-highly expressing RMSs were enriched with NK cells, which demands further study to understand the complexity of B7-H3–NK cell interaction [33].

Tumor-associated macrophages (TAMs) are another important constituent of the TME. In general, they are proven to suppress immune cytolytic function, promote tumor growth and metastases, and are usually associated with a poor prognosis in most pediatric sarcoma subtypes [85]. Activated macrophages are often classified into M1 (classical-activated) and M2 (alternative-activated) phenotypes. Generally, M1 macrophages are related to the promotion of inflammatory response against invading pathogens and tumor cells, whereas M2 macrophages tend to exert an immune-suppressive phenotype, stimulating tissue repair and tumor progression [86]. In colorectal and hepatocellular carcinoma, B7-H3 tumor expression was associated with the polarization of M1 to M2 macrophages, confirming the immunosuppressive role of B7-H3 [67,87]. However, Lavoie et al. detected that B7-H3-high expressing RMSs were enriched in M1 macrophages [33]. The positive correlation between B7-H3 expression and tumor-suppressive subtype of macrophages raises the question of determining the additional factors influencing the interaction between B7-H3 and TAM infiltration in pediatric sarcomas.

## 5. B7-H3-Related Nonimmunological Molecular Mechanisms of Carcinogenesis in Extracranial PSTs

Nonimmunological molecular mechanisms of tumorigenesis that are influenced by B7-H3 are widely examined in the tumors of adulthood [8,9,69,88]. However, there is still a lack of research regarding this field in pediatric solid cancers, and just a few studies provided results in this field [11,12,64]. Hereby, we present all the scientific data regarding B7-H3-related nonimmunological mechanisms of carcinogenesis in PSTs and denote the points that require further research.

### 5.1. Signaling Pathways

It was previously proved that B7-H3 is involved in the control of several signaling pathways including JAK/STAT, PI3K/Akt/mTOR, and Ras/Raf/MEK [9,40,41]. These singling pathways are important for various cellular processes, such as growth, proliferation, differentiation, apoptosis, embryo development, and immune system control. On the other hand, it has been observed that aberrant activation of these signaling pathways is present in many cancers, especially in chemotherapy-resistant tumors and metastatic disease [89,90,91]. Several studies indicate that B7-H3 overexpression inhibits the apoptosis of tumor cells via inadequate activation of the JAK2/STAT3 pathway [41]. The overexpression of B7-H3 in colorectal cancer has been shown to increase cancer cell migration and invasion [37] and induces resistance to apoptosis by upregulating this signaling pathway [92]. Furthermore, Liu et al. showed that B7-H3 silencing increased the sensitivity of breast cancer cell lines to chemotherapy as a consequence of inhibition of the JAK2/STAT3 signaling pathway [93]. Similarly, the studies showed that B7-H3 downregulation or inhibition can make tumor cells more sensitive to inhibitors of PI3K/Akt/mTOR and Ras/Raf/MEK signaling pathways [94]. Additionally, decreased expression of B7-H3 in breast cancer may reduce glycolytic capacity and increase cancer cell sensitivity to Akt/mTOR inhibitors [95]. Moreover, Liu et al. investigated the relationship between B7-H3 and the Ras/Raf/MEK pathway in breast cancer. They showed that overexpression of B7-H3 led to an increase in cancer stem cell pool size and promoted drug resistance via MEK activation. This correlation between B7-H3 and MEK was confirmed later in the patients’ samples. Additionally, the same group of authors showed that B7-H3 activates MEK through B7-H3–major vault protein (MVP) interaction, independent of the classical Ras-mediated pathway [96].

The role of the above-mentioned molecular pathways is also examined in pediatric tumors. It is suggested that treatments with drugs that reduce phosphorylation of the JAK/STAT pathway could decrease the viability of OS cells [97]. Moreover, Yan et al. provided strong evidence of the anti-tumor effect of JAK inhibitor AZD1480 in NB, RMS, and ES family tumors, indicating that downregulation of the JAK/STAT3 pathway could be beneficial for these patients [98]. Furthermore, mutation of RAS is observed in hematologic malignancies and several PSTs, such as PAX-fusion negative RMS, relapsed NB, melanoma, and malignant ectomesenchymoma, and several ongoing clinical trials test RAS-inhibitors in pediatric patients [99,100].

Although the abundant expression of B7-H3 in PSTs was observed by recent studies and shown to be associated with tumor aggressiveness and worse prognosis [4,11,12,64], the potential relationship between JAK2/STAT3, PI3K/Akt/mTOR, and Ras/Raf/MEK and B7-H3 in PSTs has still not been examined and further research is needed. However, a study by Kanayama et al. [64] proved that B7-H3 stimulates ARMS cell migration by regulating the expression of C-X-C chemokine receptor type 4 (CXCR4), and it was previously proved that this metastasis-associated molecule can activate different downstream signaling pathways such as JAK2/STAT3, PI3K/Akt, ERK, and NF-Κb in various cancers [101,102,103,104].

### 5.2. Matrix Metalloproteinases

Matrix metalloproteinases (MMPs) represent a family of zinc-dependent endopeptidases. The activity of these enzymes is controlled by regulatory proteins called tissue inhibitors of metalloproteinases (TIMPs), as well as by some other molecules such as α2-macroglobulin and β-Amyloid precursor protein. Although MMPs activity is an important part of cell proliferation, differentiation, angiogenesis, wound healing, and apoptosis, these enzymes are found to contribute to the pathogenesis of different types of cancer and non-cancerous diseases such as arthritis, atherosclerosis, and myocardial infarction [105,106]. The association between B7-H3 and MMP-2 and MMP-9 is well examined in some adult cancers [107,108,109]. Both the above-mentioned MMPs were shown to correlate positively with B7-H3 expression in colorectal cancer [107]. Furthermore, Tekle et al. reported that the B7-H3 knockdown resulted in reduced migration and invasion potential of melanoma cells in vitro and decreased their metastatic capacity in vivo. The silencing of B7-H3 also led to a reduced level of metastasis-associated proteins MMP-2 and Stat3, and, simultaneously, an increase in TIMP-1 and -2 levels [108]. Additionally, Xu et al. detected a positive correlation between B7-H3 and MMP-2 and revealed that the simultaneous determination of these two molecules could predict the prognosis of patients with pancreatic cancer [109]. Regarding the extracranial PSTs, B7-H3 and MMPs’ relationship was examined only in OS. Firstly, Wang et al. found that B7-H3 increased OS cell invasion. Then, they also showed that MMP-2 levels were increased in B7-H3-transfected OS cells, suggesting that B7-H3 regulates the invasion of OS cells at least partly through MMP-2 [12].

### 5.3. Cell-Cycle Control

The cell cycle represents the intracellular sequence of events, leading to cell division and the formation of two daughter cells. It comprises four phases in eukaryotes—the G1, S, G2 (together called interphase), and the M phase—mitosis [110]. The cell cycle progression from one phase to the succeeding is controlled by sequential activation and inactivation of many “checkpoints” that surveil the status of the cell and by various environmental signals. Disruption of the physiological regulation of cell-cycle progression and division is a critical event in the development of cancer [111]. B7-H3 was proven to be involved in the modulation of the cell cycle in some adult tumors [9,112].

One of the cell-cycle control mechanisms is achieved through the retinoblastoma/E2F1 (Rb/E2F1) pathway [113]. The Rb protein is a tumor suppressor that has a significant role in the negative control of the cell cycle, while its inactivation is related to the development of various types of cancer. When in a hypophosphorylated state, this protein forms a complex with E2F factors, making them unable to activate the transcription of the genes necessary for progression from the G1 to the S phase [114]. Zhang et al. examined the effect of B7-H3 knockdown on the cell cycle of human NB cells and its impact on tumor growth in vivo. Using immunohistochemical staining, they showed that B7-H3 was widely expressed in specimens of NB, and that expression was associated with poor overall survival. Afterward, they revealed that the knockdown of B7-H3 inhibited the proliferation of human NB cells in vitro and increased the accumulation of cells at the G0/G1 phase. Finally, they proved that the knockdown of B7-H3 inhibited tumor growth in vivo by upregulation of Rb and the suppression of E2F1 [11].

## 6. Targeting B7-H3—The Strategy in the Treatment of Extracranial PSTs

Many studies have provided strong evidence that B7-H3 is a valuable target protein for immune-based anti-tumor therapy due to its overexpression across different types of adult cancers but seldom in normal cells [9,16,68]. The abundant expression of B7-H3 was also found in the most common extracranial solid tumors of childhood such as NB, WT, RMS, OS, and ES [4,11,12,33], whereas its expression is absent or very low in healthy tissues and organs [16,43].

In vitro and in vivo studies showed that experimental depletion or blocking of B7-H3 in extracranial PSTs may enhance the anti-tumor immune response and inhibit tumor cell proliferation and migration [11,33,34,64]. Castriconi et al. showed that 5B14 mAb-mediated masking of 4Ig-B7-H3 molecules on the NB cell surface enhanced NK cells-mediated tumor lysis [34]. Moreover, Lavoie et al. proved that B7-H3 knockout increased T cell-mediated cytotoxicity in RMS [33]. Moreover, in the study by Kanayama et al. B7-H3 knockdown in ARMS was related to the attenuation of tumor cell migration [64]. Finally, Zhang et al. demonstrated that the knockdown of B7-H3 inhibited the proliferation of human NB cells in vitro and impaired tumor growth in vivo by inducing cell-cycle arrest [11].

Regarding the molecular mechanisms of carcinogenesis explained earlier in this review, targeting B7-H3 could be a promising strategy for the design of immunotherapeutic agents for PST treatment. The several types of targeted therapy, including mAbs (Table 1 and Table 2), chimeric antigen receptor-T (CAR-T) cell, and chimeric antigen receptor-transduced NK (CAR-NK) cell therapy (Table 3 and Table 4) underwent experimental testing in PSTs.

### 6.1. mAbs

Monoclonal antibodies are monovalent laboratory-made antibodies produced by a single B cell clone and predetermined to bind to the same epitope [130]. Different molecular mechanisms are responsible for the anti-tumor functions of mAbs. Firstly, they can enhance the anti-tumor immune response by activating Fc-mediated killing, including NK cell-ADCC, neutrophil-ADCC, complement-mediated cytotoxicity, complement-dependent cellular cytotoxicity, and antibody-dependent cell-mediated phagocytosis [3]. Moreover, mAbs can exert their role through the suppression of the tumor signaling pathways, inhibition of angiogenesis, and delivery of payloads to the targeted tumor site [131]. To become a pharmacologically active drug, mAbs can be linked to either a radioisotope (producing antibody-radioimmunoconjugates), a highly potent cytotoxic drug (making antibody-drug conjugates, ADCs), or protein toxins (giving rise to immunotoxins) [132].

Various studies investigated the role of radioimmunotherapy in PSTs, especially in NB, the most common extracranial PST. The metastatic lesions of NB are present in more than 50% of patients, being most commonly located in bone marrow, bone, and liver. Secondary lesions in CNS are rare with an overall estimated prevalence of 1.7–11.7% [133]. In 2010, Kramer et al. published the results of a clinical study testing compartmental intrathecal antibody-based radioimmunotherapy in patients with recurrent metastatic CNS NB. They showed that adding intrathecal administration of ^131^I-labeled 8H9 murine mAbs targeting B7-H3 to other therapeutic modalities, such as surgery, craniospinal irradiation, and chemotherapy could improve the survival of patients with relapsed CNS NB [134]. In 2015, Ahmed et al. performed the humanization, affinity maturation, and epitope mapping of 8H9 mAb based on structure determination, modeling, and yeast display methods [135]. Two years later, Kramer et al. published impressive results regarding the overall survival of eighty patients with NB metastatic to CNS treated with intraventricular ^131^I-8H9 in the NCT00089245 study. They found that 56% of the patients were alive at a median of 58 months of follow-up [136]. Furthermore, recently published results of a phase I clinical trial showed markedly increased survival of patients with NB metastatic to CNS treated with intraventriculary administrated ^131^I-omburtamab (8H9), compared to historical data [115]. This drug has also been studied systemically in patients with NB and CNS/leptomeningeal metastases in another ongoing open trial (NCT03275402) [116].

Another phase I trial evaluated intraperitoneal ^131^I-omburtamab in patients with desmoplastic small round cell tumor (DSRCT), a rare sarcoma of adolescents and young adults that primarily involves the peritoneum and has poor long-term survival despite aggressive multimodality treatment. In 52 tested patients (including 48 patients with DSRCT, three patients with RMS, and one with ES) investigators found no maximum tolerated dose or dose-limiting toxicities but two transient grade 4 neutropenia and one thrombocytopenia [19]. Based on the encouraging preliminary data obtained from this phase I trial and the favorable toxicity profile of the tested drug, the authors initiated a phase II trial which will examine the effect of intraperitoneal application of ^131^I-omburtamab in patients with DSRCT and other tumors with peritoneal involvement (NCT04022213) [119]. Furthermore, in a study by Modak et al., radiolabeled 8H9 showed potent anti-tumor activity in RMS xenografts, with excellent selectivity [18]. However, further clinical research regarding this agent in RMS treatment is needed.

The safety, tolerability, pharmacokinetics, pharmacodynamics, immunogenicity, and preliminary anti-tumor activity of another anti-B7-H3 mAb, enoblituzumab (MGA271), were examined in a phase I clinical trial (NCT02982941). This study was conducted on children and young adults with B7-H3-expressing relapsed or refractory malignant solid tumors including NB, RMS, OS, ES, WT, and DSRCT, but the results have not yet been published [117].

Clinical trials regarding ADCs in the treatment of malignant tumors started in the 1980s, but the results pointed to problems with drug toxicities and showed no clinical efficacy. The development of synthetic biochemistry contributed to the creation of a new generation ADCs that promise to improve tissue specificity and cytotoxicity compared to their predecessors. Each ADC consists of three main components—an antibody, a linker, and a payload, and its clinical properties depend on the characteristics of all three of these components. Nine ADCs are currently approved for cancer treatment, with many more of them being tested in preclinical and clinical studies [137]. MGC018 is a duocarmycin-based humanized ADC targeting B7-H3 that exhibited selective cytotoxicity for B7-H3-expressing tumor cells and potent in vivo activity against various adult cancer preclinical models [5]. This drug has also entered clinical studies for several adult cancers (NCT05551117, NCT05293496) [138,139]. Likewise, the results of Kurmasheva et al. suggest that MGC018 exerts profound anti-tumor activity in preclinical models of select PST models in a B7-H3-specific manner [5]. Furthermore, Kendsersky et al. investigated the activity of the B7-H3-targeting ADC m276-SL-pyrrolobenzodiazepine (m276-SL-PBD) in pediatric solid malignancy patient-derived and cell line-derived xenograft models. The m276-SL-PBD ADC showed potent anti-tumor activity against PST xenografts with limited toxicity [43].

### 6.2. CAR-T

A novel strategy to genetically modify patients’ T cells, including CAR-T cell therapy and T cell receptor (TCR) T cell therapy, has achieved substantial advances in cancer treatment. The CAR-T cell therapy begins with a collection of the patient’s blood and isolating T lymphocytes. After activation and amplification in vitro, T cells are modified by viral vector transfection to express specific CARs on the T cell surface that recognize cancer-specific antigens, which is followed by infusion back into the patient’s body. A CAR is a recombinant receptor with tumor-antigen-binding and T cell-activating functions [140]. It consists of an extracellular ligand-binding domain, a spacer (hinge) domain, a transmembrane domain, and one or more cytoplasmic domains [141,142]. The extracellular domain is a single-chain variable antibody domain (scFv), which can recognize specific tumor antigens. The hinge/transmembrane (H/Tm) domain is composed of immunoglobulin superfamily members, such as CD8, CD28, or IgG, and plays a role in signal transduction. The intracellular activatory domain of first-generation CARs is in most cases the CD3ζ chain of the TCR or the γ chain of the Fc receptor (FcRγ). Furthermore, second-generation CARs commonly include an activatory domain (CD3ζ or FcRγ) linked to costimulatory domains gained from native costimulatory molecules such as CD28 and 41BB, whereas the third-generation constructs consist of CD3ζ with two costimulatory cytoplasmic domains [140,141]. Moreover, a fourth generation of CARs is produced using further advanced genetic modifications, comprising transgenes for cytokine release and other costimulatory ligands. T lymphocytes transduced with these fourth-generation CARs are also known as T cells redirected for universal cytokine-mediated killing (TRUCKs) [143,144]. Finally, the fifth-generation CAR-T cells contain additional intracellular domains compared to their antecedents. Their CARs include truncated cytoplasmatic domains of cytokine receptors (e.g., subunit beta of interleukin-2 receptor) with a motif for binding transcription factors such as STAT-3/5. Therefore, the secreted signal makes CAR-T cells remain active and generate memory T cells, and also stimulates and reactivates the immune system [143,144].

CAR-T cell therapy showed remarkable effects in treating hematological malignancies and improved survival of patients with certain types of leukemia and lymphoma [145]. Currently, six CARs targeting CD19 or B cell maturation antigen (BCMA) are approved by the Food and Drug Administration [123]. Oppositely, the implementation of CAR-T cell therapy in solid tumors is still in the early stages of development and represents a significant challenge [145]. The factors contributing to lower CAR-T cell therapy success in solid tumors include heterogeneous expression of tumor-associated antigens (TAAs), challenges in finding tumor-specific antigens, CAR-T cell exhaustion and limited persistence, problems with penetrating physical cancer barriers and trafficking to tumor sites, and an immunosuppressive TME for CAR-T cells. Just a few TAAs such as disialoganglioside (GD2), L1 cell adhesion molecule (L1-CAM), glypican 2 (GPC2), anaplastic lymphoma kinase (ALK), neural cell adhesion molecule 1 (NCAM-1), and B7-H3 are identified as possible targets for CAR-T cell therapy in PSTs [123].

Majzner et al. tested B7-H3 CAR-T cells’ in vivo activity on xenograft models of lethal childhood cancers, including orthotopic models of ES, OS, and medulloblastoma. Systemic administration of B7-H3 CAR-T cell-mediated regression and eradication of established OS, ES, and medulloblastoma xenografts. Additionally, the authors demonstrated substantial B7-H3 CAR-T cell activity against the metastatic OS model, leading to near-complete survival of the experimental mice [4]. Similarly, the potent activity of B7-H3 CAR-T cells in controlling primary and preventing metastatic disease in the orthotopic spontaneously metastasizing xenograft model of OS was confirmed by Talbot et al. [17].

Furthermore, Nguyen et al. proved that various designs of CARs influence the anti-tumor activity of B7-H3 CAR-T cells. Firstly, they confirmed high B7-H3 expression in OS, RMS, DSRCT, NB, and malignant peripheral nerve sheath tumors [120]. However, the expression of B7-H3 in ES was low or absent [120], which is opposite to previous studies [4]. They have also performed analysis of T cells expressing B7-H3-CARs with various combinations of H/Tm (CD8α vs. CD28) and costimulatory (CD28 or 41BB) domains: CD8α/CD28, CD8α/41BB, CD28/CD28, and CD28/41BB. It was demonstrated that CAR-T cells with CD28 costimulation had superior in vitro anti-tumor effects against metastatic OS cell lines compared to those with the 41BB costimulatory domain. Additionally, it was shown that incorporating a 41BB costimulatory domain into the CD28-CAR was detrimental to effector function while expressing 41BB ligand (41BBL) on the surface of CD28-CAR-T cells was beneficial. In their in vivo study of locoregional OS and systemic lung cancer models, CD8α/CD28- and 41BBL-CAR-T cells had superior anti-tumor activity compared to other CAR-T cell populations, providing significantly better survival, with no significant differences between both constructs. In the systemic OS model, 41BBL-CAR-T cells had enhanced anti-tumor activity compared to all other CAR-T cell populations [120]. In another study, Zhang et al. constructed third-generation B7-H3 CAR-T cells that contained the B7-H3-specific scFv with a CD8 leading sequence, a CD8 H/Tm sequence, as well as the intracellular signaling domain of 41BB, CD28, and CD3ζ in tandem. The developed CAR-T cells exerted high anti-tumor efficacy in a dose-dependent manner against OS both in vitro and in vivo [45].

The dependence of CAR-T cells’ anti-tumor activity on different CAR designs was also tested by Birley et al. Firstly, they performed the screening of scFvs libraries to identify an optimal binder for B7-H3 CAR-T cells. Among seventeen candidates, the TE9 binder was proven to be the leading one. Then, the authors created the second-generation CARs. In vitro assays found that second-generation CAR-T cells with a structure TE9-CD8 H/Tm-CD28-CD3ζ had the optimal anti-tumor effects against NB cell lines. In repeat challenge and in vivo model of resistant NB, this type of B7-H3 CAR-T cell showed tumor retardation and penetrance superior to GD2 CAR-T cells recently tested in a clinical trial [121].

Moreover, the study by Du et al. showed that B7-H3 CAR-T cells effectively controlled tumor growth in metastatic xenografts of NB without causing toxicity, which potentially makes them suitable for clinical use [44]. The excellent anti-tumor activity of B7-H3 CAR-T cells against NB was also confirmed by in vitro and in vivo studies by Moghimi et al. Their experiments also detected fatal neurotoxicity in mice treated with GD2 murine CAR-T cells having the CD28 costimulatory domain, but not with those having 41BB costimulation. This confirms once again that the choice of a costimulatory domain in CAR design might play a crucial role in determining CAR-T cell effects. To reduce the possibility of systemic toxicity of GD2 CAR, the authors used a novel approach to create a synthetic Notch (SynNotch) gated CAR-T cells, GD2-B7-H3 CAR-T, recognizing GD2 as the gate and B7-H3 as the target. The SynNotch strategy enables precise control over CAR-T cell activity, leading to improved selectivity and safer treatment [122]. When the SynNotch receptor binds to a TAA, the transcriptional activator domain of the receptor is released, which induces the expression of genes coding CAR that selectively binds to the other TAA. Consequently, the expression of the second CAR is dependent on its gate, and this kind of CAR-T cell can only kill cancer cells expressing both target antigens [122,146]. The GD2-B7-H3 CAR-T cells exhibited improved specificity without causing neurotoxicity. These cells also showed a lower exhaustion profile, greater metabolic fitness, and better in vivo anti-tumor activity after repeated in vitro stimulation compared to standard B7-H3 CAR-T cells [122].

Heterogeneous expression of TAAs among cancer cells represents a major obstacle to CAR-T cell therapy success. Tian et al. examined the expression of GPC2 and B7-H3 in NB. Although GPC2 and B7-H3 mRNAs were highly expressed in NB cell lines and tumor samples in comparison to normal tissues, 32% and 18% of NB samples expressed low levels of GPC2 and B7-H3 mRNA, respectively. However, when mRNA expression of both TAAs was combined, 95% of NB samples expressed one or both molecules at a high level. The authors also found that the protein expression level of both targets correlated with mRNA levels. To overcome the heterogeneity, Tian et al. created bicistronic (BiCis) CAR-T cells targeting both B7-H3 and GPC2 [123]. Oppositely to the SynNotch system, which relies on an “AND” logic gate model created to minimize off-tumor toxicity [122], the BiCis system is based on an “OR” logic model [123]. BiCis GPC2/B7-H3 CAR-T cells were highly effective in the elimination of NB cells in vitro and in xenografts models, and exerted longer T cell persistence and greater resistance to T cell exhaustion compared with single antigen CAR-T cells [6,123].

In the past few years, the effects of B7-H3 CAR-T cells in extracranial PSTs have been tested in clinical trials NCT05562024 [124], NCT04897321 [125], NCT04483778 [126], NCT04637503 [127], NCT04864821, [128], and NCT04432649 [129], but their results have not yet been reported (Table 4).

Apart from CAR-T cell therapy, CAR-NK cells have been recognized as an attractive therapeutic option for the treatment of malignant tumors [147]. This kind of immunotherapeutic modality has shown some advantages over CAR-T cell therapy. Firstly, CAR-T cell therapy requires the use of autologous T cells due to alloreactivity and the possibility of graft-versus-host disease. On the other hand, NK cells are not activated through the MHC pathway and have a diminished risk for alloreactivity, meaning the autologous NK cells are not necessary for CAR-NK cell production. Moreover, CAR-T cell activation might lead to a massive release of inflammatory cytokines, causing cytokine release syndrome and neurotoxicity. However, the use of CAR-NK cells helps avoid these side effects because these cells are prone to produce different profiles of cytokines upon their activation compared to CAR-T cells [148]. Most CAR-NK cell studies used NK-92 cells, a continuously expanding, IL-2-dependent human NK cell line, which exerts phenotypic and functional features of activated NK cells, except the expression of CD16 (FcγRIII). Grote et al. proved that B7-H3 CAR-NK-92 cells exerted potent anti-tumor activity against NB cell lines in vitro, without unwanted off-target cytotoxic effects. Moreover, B7-H3 CAR-NK-92 cells exhibited increased cytotoxicity in a three-dimensional NB spheroid model. This kind of laboratory model bridges the gap between in vitro and in vivo models by simulating in vivo morphology, polarity, cell connectivity, gene expression, and tissue architecture. Finally, B7-H3 CAR-NK-92 cells produced multiple NK effector molecules as well as immunity-stimulating cytokines [46].

## 7. Conclusions

In the past decade, immunotherapy significantly improved the survival of adult patients with various solid cancers. However, being considered immunologically “cold”, PSTs were far less responsive to modern immunotherapeutic modalities. In recent years, research on childhood cancers has identified B7-H3 as a molecule possessing the great potential to become a leading therapeutic target. This molecule is abundantly expressed on PSTs and minimally expressed or absent on normal tissues making it ideal for limiting off-target drug toxicity.

The advances in synthetic biochemistry facilitated the construction of various immunotherapeutic agents and their testing in preclinical settings. B7-H3-targeting ADCs exhibited profound anti-tumor effects against preclinical models of pediatric solid malignancies. Furthermore, the remarkable anti-tumor activity of B7-H3 CAR-T cells in PSTs was observed by in vitro and in vivo studies. We emphasize that the proper design of CAR is a key point for CAR-T cells’ effectiveness. It was demonstrated that the choice of CAR’s costimulatory domain significantly influences its properties. Additionally, combined targeting of B7-H3 and some other TAAs, such as GD2 or GPC2, by CAR-T cells provided excellent preclinical results. Designing the dual targeting CARs for PSTs immunotherapy could limit the off-tumor toxicity or help to overcome the problems with the heterogeneous expression of TAAs. Moreover, the development of B7-H3 targeting CAR-NK cells has become an attractive alternative for dealing with some weaknesses of CAR-T cells, such as therapy-induced side effects.

Finally, the results of clinical trials show that B7-H3 mAbs linked to radioisotopes exert potent anti-tumor activity and have a favorable toxicity profile in some PSTs. Although there have not been enough published results of clinical trials regarding B7-H3 as an immunotherapeutic target in extracranial solid cancers of childhood, the amount of clinical research in this field has significantly increased in recent years. In the future, it is crucial to precisely determine the molecular mechanisms of B7-H3 tumor-promoting activity in extracranial PSTs. This will help us understand the possible ways to impair the effects of this checkpoint molecule. Ultimately, it will ease the creation of effective immunotherapy-based drugs which could be used to treat refractory and metastatic PSTs.

## Figures and Tables

**Table 1:** B7-H3-targeting mAb-based anti-tumor agents in preclinical studies involving extracranial PSTs.

Study (Reference)	Agent/Drug	Route of Administration	Study Models
Modak et al. [18]	^131^I-labeled 8H9 mAbs ^1^	Intravenous	Cell line-derived RMS xenograft model
Kurmasheva et al. [5]	MGC018 ADCs	Intraperitoneal	Patient-derived NB, OS, ES, and RMS xenograft models
Kendsersky et al. [43]	m276-SL-pyrrolobenzodiazepine ADCs	Intraperitoneal	Patient-derived and cell line-derived PSTs xenograft models

^1^ 8H9 mAb—a B7-H3-targeting mAb also known as omburtamab. Abbreviations: B7-H3: B7 homolog 3; mAb: monoclonal antibody; PSTs: pediatric solid tumors; RMS: rhabdomyosarcoma; ADCs: antibody-drug conjugates; NB: neuroblastoma; OS: osteosarcoma; ES: Ewing sarcoma; RMS: rhabdomyosarcoma.

**Table 2:** B7-H3-targeting mAb-based anti-tumor agents in clinical studies involving PSTs. The table shows the studies on extracranial PSTs as well as the studies involving their metastases in the CNS.

Study/Phase (Reference)	Agent/Drug	Route of Administration	Study Participants	Status
NCT00089245 Phase I [115]	^131^I-labeled 8H9 mAbs ^1^	Intrathecal	Patients with refractory, recurrent, or advanced CNS or leptomeningeal cancer	Active, not recruiting
NCT03275402 Phase II/III [116]	^131^I-labeled 8H9 mAbs	Intraventricular	Patients with NB metastatic to CNS or with leptomeningeal metastases	Recruiting
NCT02982941 Phase I [117]	Enoblituzumab ^2^	Intravenous	Patients with relapsed or refractory NB, RMS, OS, ES, WT, or DSRCT	Completed
NCT05064306 N/A [118]	^131^I-omburtamab	Intraventricular	Patients with solid tumors and CNS/leptomeningeal involvement	Available
NCT04022213 Phase II [119]	^131^I-omburtamab	Intraperitoneal	DSRCT and other tumors with peritoneal involvement	Recruiting

^1^ 8H9 mAb—a B7-H3-targeting mAb also known as omburtamab. ^2^ Enoblituzumab—a B7-H3-targeting mAb also known as MGA271. Abbreviations: B7-H3: B7 homolog 3; mAb: monoclonal antibody; PSTs: pediatric solid tumors; CNS: the central nervous system; NB: neuroblastoma; RMS: rhabdomyosarcoma; OS: osteosarcoma; ES: Ewing sarcoma; WT: Wilms tumor; DSRCT: desmoplastic small round cell tumor.

**Table 3:** B7-H3-targeting CAR-T cells in preclinical studies involving extracranial PSTs.

Study (Reference)	Agent/Drug	Study Models
Majzner et al. [4]	B7-H3 CAR-T cells	- Orthotopic xenograft models of ES and OS - Metastatic OS xenograft model
Talbot et al. [17]	B7-H3 CAR-T cells	- In vitro OS model - Orthotopic spontaneously metastasizing OS xenograft model
Nguyen et al. [120]	B7-H3 CAR-T cells	- In vitro OS model - Locoregional OS xenograft model - Systemic OS xenograft model
Zhang et. al. [45]	B7-H3 CAR-T cells	- In vitro OS model - Patient-derived xenograft model of OS
Birley et al. [121]	B7-H3 CAR-T cells	- In vitro NB model - In vivo NB model
Du et al. [44]	B7-H3 CAR-T cells	- In vitro NB model - Metastatic NB xenograft model
Moghimi et al. [122]	GD2-B7-H3 CAR-T cells	- In vitro NB model - Metastatic NB xenograft model
Tian et al. [123]	BiCis GPC2/B7-H3 CAR-T cells	- In vitro NB model - Patient-derived xenograft model of NB
Grote et al. [46]	B7-H3 CAR-NK-92	- In vitro NB model - Three-dimensional NB spheroid model

Abbreviations: B7-H3: B7 homolog 3; CAR: chimeric antigen receptor; PSTs: pediatric solid tumors; ES: Ewing sarcoma; OS: osteosarcoma; NB: neuroblastoma; GD2: disialoganglioside; BiCis: bicistronic; GPC2: glypican 2; NK: natural killer.

**Table 4:** B7-H3-targeting CAR-T cells in clinical studies involving PSTs. The table shows the studies on extracranial PSTs as well as the studies involving their metastases in CNS.

Study/Phase (Reference)	Agent/Drug	Study Participants	Status
NCT05562024 Phase I [124]	B7-H3 CAR-T cells TAA06	Patients with relapsed/refractory NB	Recruiting
NCT04897321 Phase I [125]	B7-H3 CAR-T cells	Patients with OS, RMS, ES, NB, WT, DSRCT, and other B7-H3-expressing PSTs	Recruiting
NCT04483778 Phase I [126]	41BBζ B7-H3-EGFRt-DHFR CAR-T cells	Children and young adults with relapsed or refractory non-CNS solid tumors	Recruiting
NCT04637503 Phase I/II [127]	4SCAR-T cells targeting GD2, PSMA and B7-H3	Patients with relapsed/refractory NB	Recruiting
NCT04864821 Early Phase I [128]	B7-H3 CAR-T cells	Patients with NB and OS	Not yet recruiting
NCT04432649 Phase I/II [129]	4SCAR-T cells targeting B7-H3	Patients with refractory and/or recurrent solid tumors	Recruiting

Abbreviations: B7-H3: B7 homolog 3; CAR: chimeric antigen receptor; PSTs: pediatric solid tumors; CNS: central nervous system; NB: neuroblastoma; OS: osteosarcoma; RMS: rhabdomyosarcoma; ES: Ewing sarcoma; WT: Wilms tumor; DSRCT desmoplastic small round cell tumor; EGFRt: truncated epidermal growth factor receptor; DHFR: dihydrofolate reductase; 4SCAR: fourth-generation CAR.

## Data Availability

Not applicable.

## References

[1] World Health Organization (2021). CureAll Framework: WHO Global Initiative for Childhood Cancer: Increasing Access, Advancing Quality, Saving Lives.

[2] Ring E.K., Markert J.M., Gillespie G.Y., Friedman G.K. (2017). Checkpoint Proteins in Pediatric Brain and Extracranial Solid Tumors: Opportunities for Immunotherapy. Clin. Cancer Res..

[3] Casey D.L., Cheung N.V. (2020). Immunotherapy of Pediatric Solid Tumors: Treatments at a Crossroads, with an Emphasis on Antibodies. Cancer Immunol. Res..

[4] Majzner R.G., Theruvath J.L., Nellan A., Heitzeneder S., Cui Y., Mount C.W., Rietberg S.P., Linde M.H., Xu P., Rota C. (2019). CAR T Cells Targeting B7-H3, a Pan-Cancer Antigen, Demonstrate Potent Preclinical Activity Against Pediatric Solid Tumors and Brain Tumors. Clin. Cancer Res..

[5] Kurmasheva R., Mosse Y.P., Pozo V.D., Earley E.J., Erickson S.W., Groff D., Kolb E.A., Krytska K., Smith M.A., Tsang M. (2021). Testing of B7-H3 targeting antibody-drug conjugate (ADC) MGC018 in models of pediatric solid tumors by the Pediatric Preclinical Testing Consortium (PPTC). J. Clin. Oncol..

[6] Pulido R., Nunes-Xavier C.E. (2022). Hopes on immunotherapy targeting B7-H3 in neuroblastoma. Transl. Oncol..

[7] Vitanza N.A., Wilson A.L., Huang W., Seidel K., Brown C., Gustafson J.A., Yokoyama J.K., Johnson A.J., Baxter B.A., Koning R.W. (2023). Intraventricular B7-H3 CAR T Cells for Diffuse Intrinsic Pontine Glioma: Preliminary First-in-Human Bioactivity and Safety. Cancer Discov..

[8] Kontos F., Michelakos T., Kurokawa T., Sadagopan A., Schwab J.H., Ferrone C.R., Ferrone S. (2021). B7-H3: An attractive target for antibody-based immunotherapy. Clin. Cancer Res..

[9] Rasic P., Jovanovic-Tucovic M., Jeremic M., Djuricic S.M., Vasiljevic Z.V., Milickovic M., Savic D. (2021). B7 homologue 3 as a prognostic biomarker and potential therapeutic target in gastrointestinal tumors. World J. Gastrointest. Oncol..

[10] Gregorio A., Corrias M.V., Castriconi R., Dondero A., Mosconi M., Gambini C., Moretta A., Moretta L., Bottino C. (2008). Small round blue cell tumours: Diagnostic and prognostic usefulness of the expression of B7-H3 surface molecule. Histopathology.

[11] Zhang H., Zhang J., Li C., Xu H., Dong R., Chen C.C., Hua W. (2020). Survival Association and Cell Cycle Effects of B7H3 in Neuroblastoma. J. Korean Neurosurg. Soc..

[12] Wang L., Zhang Q., Chen W., Shan B., Ding Y., Zhang G., Cao N., Liu L., Zhang Y. (2013). B7-H3 is overexpressed in patients suffering osteosarcoma and associated with tumor aggressiveness and metastasis. PLoS ONE.

[13] Haydar D., Houke H., Chiang J., Yi Z., Ode Z., Caldwell K., Zhu X., Mercer K.S., Stripay J.L., Shaw T.I. (2021). Cell-surface antigen profiling of pediatric brain tumors: B7-H3 is consistently expressed and can be targeted via local or systemic CAR T-cell delivery. Neuro Oncol..

[14] Maachani U.B., Tosi U., Pisapia D.J., Mukherjee S., Marnell C.S., Voronina J., Martinez D., Santi M., Dahmane N., Zhou Z. (2020). B7-H3 as a Prognostic Biomarker and Therapeutic Target in Pediatric central nervous system Tumors. Transl. Oncol..

[15] Li S., Poolen G.C., van Vliet L.C., Schipper J.G., Broekhuizen R., Monnikhof M., Van Hecke W., Vermeulen J.F., Bovenschen N. (2022). Pediatric medulloblastoma express immune checkpoint B7-H3. Clin. Transl. Oncol..

[16] Zhang Z., Jiang C., Liu Z., Yang M., Tang X., Wang Y., Zheng M., Huang J., Zhong K., Zhao S. (2020). B7-H3-Targeted CAR-T Cells Exhibit Potent Antitumor Effects on Hematologic and Solid Tumors. Mol. Ther. Oncolytics.

[17] Talbot L.J., Chabot A., Funk A., Nguyen P., Wagner J., Ross A., Tillman H., Davidoff A., Gottschalk S., DeRenzo C. (2021). A Novel Orthotopic Implantation Technique for Osteosarcoma Produces Spontaneous Metastases and Illustrates Dose-Dependent Efficacy of B7-H3-CAR T Cells. Front. Immunol..

[18] Modak S., Guo H.F., Humm J.L., Smith-Jones P.M., Larson S.M., Cheung N.K. (2005). Radioimmunotargeting of human rhabdomyosarcoma using monoclonal antibody 8H9. Cancer Biother. Radiopharm..

[19] Modak S., Zanzonico P., Grkovski M., Slotkin E.K., Carrasquillo J.A., Lyashchenko S.K., Lewis J.S., Cheung I.Y., Heaton T., LaQuaglia M.P. (2020). B7H3-Directed Intraperitoneal Radioimmunotherapy with Radioiodinated Omburtamab for Desmoplastic Small Round Cell Tumor and Other Peritoneal Tumors: Results of a Phase I Study. J. Clin. Oncol..

[20] Li G., Quan Y., Che F., Wang L. (2018). B7-H3 in tumors: Friend or foe for tumor immunity?. Cancer Chemother. Pharmacol..

[21] Picarda E., Ohaegbulam K.C., Zang X. (2016). Molecular Pathways: Targeting B7-H3 (CD276) for Human Cancer Immunotherapy. Clin. Cancer Res..

[22] Xu H., Cheung I.Y., Guo H.F., Cheung N.K. (2009). MicroRNA miR-29 modulates expression of immunoinhibitory molecule B7-H3: Potential implications for immune based therapy of human solid tumors. Cancer Res..

[23] Yang X., Feng K.X., Li H., Wang L., Xia H. (2020). MicroRNA-199a Inhibits Cell Proliferation, Migration, and Invasion and Activates AKT/mTOR Signaling Pathway by Targeting B7-H3 in Cervical Cancer. Technol. Cancer Res. Treat..

[24] Wang L., Kang F.B., Sun N., Wang J., Chen W., Li D., Shan B.E. (2016). The tumor suppressor miR-124 inhibits cell proliferation and invasion by targeting B7-H3 in osteosarcoma. Tumour. Biol..

[25] Inamura K., Takazawa Y., Inoue Y., Yokouchi Y., Kobayashi M., Saiura A., Shibutani T., Ishikawa Y. (2018). Tumor B7-H3 (CD276) Expression and Survival in Pancreatic Cancer. J. Clin. Med..

[26] Zheng Y., Liao N., Wu Y., Gao J., Li Z., Liu W., Wang Y., Li M., Li X., Chen L. (2019). High expression of B7H2 or B7H3 is associated with poor prognosis in hepatocellular carcinoma. Mol. Med. Rep..

[27] Mao Y., Li W., Chen K., Xie Y., Liu Q., Yao M., Duan W., Zhou X., Liang R., Tao M. (2015). B7-H1 and B7-H3 are independent predictors of poor prognosis in patients with non-small cell lung cancer. Oncotarget.

[28] Cong F., Yu H., Gao X. (2017). Expression of CD24 and B7-H3 in breast cancer and the clinical significance. Oncol. Lett..

[29] Zang X., Sullivan P.S., Soslow R.A., Waitz R., Reuter V.E., Wilton A., Thaler H.T., Arul M., Slovin S.F., Wei J. (2010). Tumor associated endothelial expression of B7-H3 predicts survival in ovarian carcinomas. Mod. Pathol..

[30] Amori G., Sugawara E., Shigematsu Y., Akiya M., Kunieda J., Yuasa T., Yamamoto S., Yonese J., Takeuchi K., Inamura K. (2021). Tumor B7-H3 expression in diagnostic biopsy specimens and survival in patients with metastatic prostate cancer. Prostate Cancer Prostatic Dis..

[31] Saeednejad Zanjani L., Madjd Z., Axcrona U., Abolhasani M., Rasti A., Asgari M., Fodstad O., Andersson Y. (2020). Cytoplasmic expression of B7-H3 and membranous EpCAM expression are associated with higher grade and survival outcomes in patients with clear cell renal cell carcinoma. Ann. Diagn. Pathol..

[32] Lin W., Xu Y., Gao J., Zhang H., Sun Y., Qiu X., Huang Q., Kong L., Lu J.J. (2021). Multi-Omics Data Analyses Identify B7-H3 as a Novel Prognostic Biomarker and Predict Response to Immune Checkpoint Blockade in Head and Neck Squamous Cell Carcinoma. Front. Immunol..

[33] Lavoie R.R., Gargollo P.C., Ahmed M.E., Kim Y., Baer E., Phelps D.A., Charlesworth C.M., Madden B.J., Wang L., Houghton P.J. (2021). Surfaceome Profiling of Rhabdomyosarcoma Reveals B7-H3 as a Mediator of Immune Evasion. Cancers.

[34] Castriconi R., Dondero A., Augugliaro R., Cantoni C., Carnemolla B., Sementa A.R., Negri F., Conte R., Corrias M.V., Moretta L. (2004). Identification of 4Ig-B7-H3 as a neuroblastoma-associated molecule that exerts a protective role from an NK cell-mediated lysis. Proc. Natl. Acad. Sci. USA.

[35] Chapoval A.I., Ni J., Lau J.S., Wilcox R.A., Flies D.B., Liu D., Dong H., Sica G.L., Zhu G., Tamada K. (2001). B7-H3: A costimulatory molecule for T cell activation and IFN-gamma production. Nat. Immunol..

[36] Castellanos J.R., Purvis I.J., Labak C.M., Guda M.R., Tsung A.J., Velpula K.K., Asuthkar S. (2017). B7-H3 role in the immune landscape of cancer. Am. J. Clin. Exp. Immunol..

[37] Liu F., Zhang T., Zou S., Jiang B., Hua D. (2015). B7H3 promotes cell migration and invasion through the Jak2/Stat3/MMP9 signaling pathway in colorectal cancer. Mol. Med. Rep..

[38] Wang R., Ma Y., Zhan S., Zhang G., Cao L., Zhang X., Shi T., Chen W. (2020). B7-H3 promotes colorectal cancer angiogenesis through activating the NF-kappaB pathway to induce VEGFA expression. Cell Death Dis..

[39] Ma Y., Zhan S., Lu H., Wang R., Xu Y., Zhang G., Cao L., Shi T., Zhang X., Chen W. (2020). B7-H3 regulates KIF15-activated ERK1/2 pathway and contributes to radioresistance in colorectal cancer. Cell Death Dis..

[40] Zhou W.T., Jin W.L. (2021). B7-H3/CD276: An Emerging Cancer Immunotherapy. Front. Immunol..

[41] Zhou X., Ouyang S., Li J., Huang X., Ai X., Zeng Y., Lv Y., Cai M. (2019). The novel non-immunological role and underlying mechanisms of B7-H3 in tumorigenesis. J. Cell. Physiol..

[42] Zhang X., Fang C., Zhang G., Jiang F., Wang L., Hou J. (2017). Prognostic value of B7-H3 expression in patients with solid tumors: A meta-analysis. Oncotarget.

[43] Kendsersky N.M., Lindsay J., Kolb E.A., Smith M.A., Teicher B.A., Erickson S.W., Earley E.J., Mosse Y.P., Martinez D., Pogoriler J. (2021). The B7-H3-Targeting Antibody-Drug Conjugate m276-SL-PBD Is Potently Effective Against Pediatric Cancer Preclinical Solid Tumor Models. Clin. Cancer Res..

[44] Du H., Hirabayashi K., Ahn S., Kren N.P., Montgomery S.A., Wang X., Tiruthani K., Mirlekar B., Michaud D., Greene K. (2019). Antitumor Responses in the Absence of Toxicity in Solid Tumors by Targeting B7-H3 via Chimeric Antigen Receptor T Cells. Cancer Cell.

[45] Zhang Q., Zhang Z., Liu G., Li D., Gu Z., Zhang L., Pan Y., Cui X., Wang L., Liu G. (2022). B7-H3 targeted CAR-T cells show highly efficient anti-tumor function against osteosarcoma both in vitro and in vivo. BMC Cancer.

[46] Grote S., Chan K.C.-H., Baden C., Bösmüller H., Sulyok M., Frauenfeld L., Ebinger M., Handgretinger R., Schleicher S. (2021). CD276 as a novel CAR NK-92 therapeutic target for neuroblastoma. Adv. Cell Gene Ther..

[47] Medzhitov R., Horng T. (2009). Transcriptional control of the inflammatory response. Nat. Rev. Immunol..

[48] Carpenter S., Ricci E.P., Mercier B.C., Moore M.J., Fitzgerald K.A. (2014). Post-transcriptional regulation of gene expression in innate immunity. Nat. Rev. Immunol..

[49] Jacobs E., Mills J.D., Janitz M. (2012). The role of RNA structure in posttranscriptional regulation of gene expression. J. Genet. Genom..

[50] Corbett A.H. (2018). Post-transcriptional regulation of gene expression and human disease. Curr. Opin. Cell Biol..

[51] Bartel D.P. (2009). MicroRNAs: Target recognition and regulatory functions. Cell.

[52] Felekkis K., Touvana E., Stefanou C., Deltas C. (2010). microRNAs: A newly described class of encoded molecules that play a role in health and disease. Hippokratia.

[53] Shenouda S.K., Alahari S.K. (2009). MicroRNA function in cancer: Oncogene or a tumor suppressor?. Cancer Metastasis Rev..

[54] O’Brien J., Hayder H., Zayed Y., Peng C. (2018). Overview of MicroRNA Biogenesis, Mechanisms of Actions, and Circulation. Front. Endocrinol..

[55] Saliminejad K., Khorram Khorshid H.R., Soleymani Fard S., Ghaffari S.H. (2019). An overview of microRNAs: Biology, functions, therapeutics, and analysis methods. J. Cell Physiol..

[56] Hammond S.M. (2015). An overview of microRNAs. Adv. Drug Deliv. Rev..

[57] Lu J., Getz G., Miska E.A., Alvarez-Saavedra E., Lamb J., Peck D., Sweet-Cordero A., Ebert B.L., Mak R.H., Ferrando A.A. (2005). MicroRNA expression profiles classify human cancers. Nature.

[58] Esquela-Kerscher A., Slack F.J. (2006). Oncomirs-microRNAs with a role in cancer. Nat. Rev. Cancer.

[59] Nygren M.K., Tekle C., Ingebrigtsen V.A., Makela R., Krohn M., Aure M.R., Nunes-Xavier C.E., Perala M., Tramm T., Alsner J. (2014). Identifying microRNAs regulating B7-H3 in breast cancer: The clinical impact of microRNA-29c. Br. J. Cancer.

[60] Wang J., Chen X., Xie C., Sun M., Hu C., Zhang Z., Luan L., Zhou J., Zhou J., Zhu X. (2021). MicroRNA miR-29a Inhibits Colon Cancer Progression by Downregulating B7-H3 Expression: Potential Molecular Targets for Colon Cancer Therapy. Mol. Biotechnol..

[61] Cheung I.Y., Farazi T.A., Ostrovnaya I., Xu H., Tran H., Mihailovic A., Tuschl T., Cheung N.K. (2014). Deep MicroRNA sequencing reveals downregulation of miR-29a in neuroblastoma central nervous system metastasis. Genes Chromosomes Cancer.

[62] Zhou W.Y., Cai Z.R., Liu J., Wang D.S., Ju H.Q., Xu R.H. (2020). Circular RNA: Metabolism, functions and interactions with proteins. Mol. Cancer.

[63] Wang L., Zhang G.C., Kang F.B., Zhang L., Zhang Y.Z. (2019). hsa_circ0021347 as a Potential Target Regulated by B7-H3 in Modulating the Malignant Characteristics of Osteosarcoma. BioMed Res. Int..

[64] Kanayama T., Miyachi M., Sugimoto Y., Yagyu S., Kikuchi K., Tsuchiya K., Iehara T., Hosoi H. (2021). Reduced B7-H3 expression by PAX3-FOXO1 knockdown inhibits cellular motility and promotes myogenic differentiation in alveolar rhabdomyosarcoma. Sci. Rep..

[65] Trubicka J., Grajkowska W., Dembowska-Baginska B. (2022). Molecular Markers of Pediatric Solid Tumors-Diagnosis, Optimizing Treatments, and Determining Susceptibility: Current State and Future Directions. Cells.

[66] Azorsa D.O., Bode P.K., Wachtel M., Cheuk A.T.C., Meltzer P.S., Vokuhl C., Camenisch U., Khov H.L., Bode B., Schafer B.W. (2021). Immunohistochemical detection of PAX-FOXO1 fusion proteins in alveolar rhabdomyosarcoma using breakpoint specific monoclonal antibodies. Mod. Pathol..

[67] Kang F.B., Wang L., Li D., Zhang Y.G., Sun D.X. (2015). Hepatocellular carcinomas promote tumor-associated macrophage M2-polarization via increased B7-H3 expression. Oncol. Rep..

[68] Yang S., Wei W., Zhao Q. (2020). B7-H3, a checkpoint molecule, as a target for cancer immunotherapy. Int. J. Biol. Sci..

[69] Li Y., Zhang J., Han S., Qian Q., Chen Q., Liu L., Zhang Y. (2017). B7-H3 promotes the proliferation, migration and invasiveness of cervical cancer cells and is an indicator of poor prognosis. Oncol. Rep..

[70] Li Y., Guo G., Song J., Cai Z., Yang J., Chen Z., Wang Y., Huang Y., Gao Q. (2017). B7-H3 Promotes the Migration and Invasion of Human Bladder Cancer Cells via the PI3K/Akt/STAT3 Signaling Pathway. J. Cancer.

[71] Yin S.J., Wang W.J., Zhang J.Y. (2015). Expression of B7-H3 in cancer tissue during osteosarcoma progression in nude mice. Genet. Mol. Res..

[72] Terry R.L., Meyran D., Ziegler D.S., Haber M., Ekert P.G., Trapani J.A., Neeson P.J. (2020). Immune profiling of pediatric solid tumors. J. Clin. Investig..

[73] Fridman W.H., Zitvogel L., Sautes-Fridman C., Kroemer G. (2017). The immune contexture in cancer prognosis and treatment. Nat. Rev. Clin. Oncol..

[74] Sun J., Chen L.J., Zhang G.B., Jiang J.T., Zhu M., Tan Y., Wang H.T., Lu B.F., Zhang X.G. (2010). Clinical significance and regulation of the costimulatory molecule B7-H3 in human colorectal carcinoma. Cancer Immunol. Immunother..

[75] Guo L., Liu Z., Zhang Y., Quan Q., Huang L., Xu Y., Cao L., Zhang X. (2019). Association of increased B7 protein expression by infiltrating immune cells with progression of gastric carcinogenesis. Medicine.

[76] Chen L., Chen J., Xu B., Wang Q., Zhou W., Zhang G., Sun J., Shi L., Pei H., Wu C. (2015). B7-H3 expression associates with tumor invasion and patient’s poor survival in human esophageal cancer. Am. J. Transl. Res..

[77] Wang L., Cao N.N., Wang S., Man H.W., Li P.F., Shan B.E. (2016). Roles of coinhibitory molecules B7-H3 and B7-H4 in esophageal squamous cell carcinoma. Tumour. Biol..

[78] Kim N.I., Park M.H., Kweon S.S., Lee J.S. (2020). B7-H3 and B7-H4 Expression in Breast Cancer and Their Association with Clinicopathological Variables and T Cell Infiltration. Pathobiology.

[79] Wienke J., Dierselhuis M.P., Tytgat G.A.M., Kunkele A., Nierkens S., Molenaar J.J. (2021). The immune landscape of neuroblastoma: Challenges and opportunities for novel therapeutic strategies in pediatric oncology. Eur. J. Cancer.

[80] Zhou Q., Li K., Lai Y., Yao K., Wang Q., Zhan X., Peng S., Cai W., Yao W., Zang X. (2021). B7 score and T cell infiltration stratify immune status in prostate cancer. J. Immunother. Cancer.

[81] Li C., Jiang P., Wei S., Xu X., Wang J. (2020). Regulatory T cells in tumor microenvironment: New mechanisms, potential therapeutic strategies and future prospects. Mol. Cancer.

[82] Jin Y., Zhang P., Li J., Zhao J., Liu C., Yang F., Yang D., Gao A., Lin W., Ma X. (2015). B7-H3 in combination with regulatory T cell is associated with tumor progression in primary human non-small cell lung cancer. Int. J. Clin. Exp. Pathol..

[83] Jewett A., Kos J., Kaur K., Safaei T., Sutanto C., Chen W., Wong P., Namagerdi A.K., Fang C., Fong Y. (2020). Natural Killer Cells: Diverse Functions in Tumor Immunity and Defects in Pre-neoplastic and Neoplastic Stages of Tumorigenesis. Mol. Ther. Oncolytics.

[84] Bottino C., Dondero A., Bellora F., Moretta L., Locatelli F., Pistoia V., Moretta A., Castriconi R. (2014). Natural killer cells and neuroblastoma: Tumor recognition, escape mechanisms, and possible novel immunotherapeutic approaches. Front. Immunol..

[85] Koo J., Hayashi M., Verneris M.R., Lee-Sherick A.B. (2020). Targeting Tumor-Associated Macrophages in the Pediatric Sarcoma Tumor Microenvironment. Front. Oncol..

[86] Lin Y., Xu J., Lan H. (2019). Tumor-associated macrophages in tumor metastasis: Biological roles and clinical therapeutic applications. J. Hematol. Oncol..

[87] Mao Y., Chen L., Wang F., Zhu D., Ge X., Hua D., Sun J. (2017). Cancer cell-expressed B7-H3 regulates the differentiation of tumor-associated macrophages in human colorectal carcinoma. Oncol. Lett..

[88] Dong P., Xiong Y., Yue J., Hanley S.J.B., Watari H. (2018). B7H3 As a Promoter of Metastasis and Promising Therapeutic Target. Front. Oncol..

[89] Mengie Ayele T., Tilahun Muche Z., Behaile Teklemariam A., Bogale Kassie A., Chekol Abebe E. (2022). Role of JAK2/STAT3 Signaling Pathway in the Tumorigenesis, Chemotherapy Resistance, and Treatment of Solid Tumors: A Systemic Review. J. Inflamm. Res..

[90] Xia P., Xu X.Y. (2015). PI3K/Akt/mTOR signaling pathway in cancer stem cells: From basic research to clinical application. Am. J. Cancer Res..

[91] McCubrey J.A., Steelman L.S., Chappell W.H., Abrams S.L., Wong E.W., Chang F., Lehmann B., Terrian D.M., Milella M., Tafuri A. (2007). Roles of the Raf/MEK/ERK pathway in cell growth, malignant transformation and drug resistance. Biochim. Biophys. Acta.

[92] Zhang T., Jiang B., Zou S.T., Liu F., Hua D. (2015). Overexpression of B7-H3 augments anti-apoptosis of colorectal cancer cells by Jak2-STAT3. World J. Gastroenterol..

[93] Liu H., Tekle C., Chen Y.W., Kristian A., Zhao Y., Zhou M., Liu Z., Ding Y., Wang B., Maelandsmo G.M. (2011). B7-H3 silencing increases paclitaxel sensitivity by abrogating Jak2/Stat3 phosphorylation. Mol. Cancer Ther..

[94] Lin Z., Wu Z., Luo W. (2021). A Novel Treatment for Ewing’s Sarcoma: Chimeric Antigen Receptor-T Cell Therapy. Front. Immunol..

[95] Nunes-Xavier C.E., Karlsen K.F., Tekle C., Pedersen C., Oyjord T., Hongisto V., Nesland J.M., Tan M., Sahlberg K.K., Fodstad O. (2016). Decreased expression of B7-H3 reduces the glycolytic capacity and sensitizes breast cancer cells to AKT/mTOR inhibitors. Oncotarget.

[96] Liu Z., Zhang W., Phillips J.B., Arora R., McClellan S., Li J., Kim J.H., Sobol R.W., Tan M. (2019). Immunoregulatory protein B7-H3 regulates cancer stem cell enrichment and drug resistance through MVP-mediated MEK activation. Oncogene.

[97] Sandoval-Usme M.C., Umana-Perez A., Guerra B., Hernandez-Perera O., Garcia-Castellano J.M., Fernandez-Perez L., Sanchez-Gomez M. (2014). Simvastatin impairs growth hormone-activated signal transducer and activator of transcription (STAT) signaling pathway in UMR-106 osteosarcoma cells. PLoS ONE.

[98] Yan S., Li Z., Thiele C.J. (2013). Inhibition of STAT3 with orally active JAK inhibitor, AZD1480, decreases tumor growth in Neuroblastoma and Pediatric Sarcomas In vitro and In vivo. Oncotarget.

[99] Vaseva A.V., Yohe M.E. (2020). Targeting RAS in pediatric cancer: Is it becoming a reality?. Curr. Opin. Pediatr..

[100] Eleveld T.F., Oldridge D.A., Bernard V., Koster J., Colmet Daage L., Diskin S.J., Schild L., Bentahar N.B., Bellini A., Chicard M. (2015). Relapsed neuroblastomas show frequent RAS-MAPK pathway mutations. Nat. Genet..

[101] Chatterjee S., Behnam Azad B., Nimmagadda S. (2014). The intricate role of CXCR4 in cancer. Adv. Cancer Res..

[102] Liu X., Xiao Q., Bai X., Yu Z., Sun M., Zhao H., Mi X., Wang E., Yao W., Jin F. (2014). Activation of STAT3 is involved in malignancy mediated by CXCL12-CXCR4 signaling in human breast cancer. Oncol. Rep..

[103] Li Y., Yang X., Wu Y., Zhao K., Ye Z., Zhu J., Xu X., Zhao X., Xing C. (2017). B7-H3 promotes gastric cancer cell migration and invasion. Oncotarget.

[104] Kukreja P., Abdel-Mageed A.B., Mondal D., Liu K., Agrawal K.C. (2005). Up-regulation of CXCR4 expression in PC-3 cells by stromal-derived factor-1alpha (CXCL12) increases endothelial adhesion and transendothelial migration: Role of MEK/ERK signaling pathway-dependent NF-kappaB activation. Cancer Res..

[105] Bassiouni W., Ali M.A.M., Schulz R. (2021). Multifunctional intracellular matrix metalloproteinases: Implications in disease. FEBS J..

[106] Murphy G., Knauper V., Atkinson S., Butler G., English W., Hutton M., Stracke J., Clark I. (2002). Matrix metalloproteinases in arthritic disease. Arthritis Res. Ther..

[107] Jiang B., Zhang T., Liu F., Sun Z., Shi H., Hua D., Yang C. (2016). The co-stimulatory molecule B7-H3 promotes the epithelial-mesenchymal transition in colorectal cancer. Oncotarget.

[108] Tekle C., Nygren M.K., Chen Y.W., Dybsjord I., Nesland J.M., Maelandsmo G.M., Fodstad O. (2012). B7-H3 contributes to the metastatic capacity of melanoma cells by modulation of known metastasis-associated genes. Int. J. Cancer.

[109] Xu L., Ding X., Tan H., Qian J. (2013). Correlation between B7-H3 expression and matrix metalloproteinases 2 expression in pancreatic cancer. Cancer Cell Int..

[110] Wang Z. (2021). Regulation of Cell Cycle Progression by Growth Factor-Induced Cell Signaling. Cells.

[111] Meeran S.M., Katiyar S.K. (2008). Cell cycle control as a basis for cancer chemoprevention through dietary agents. Front. Biosci..

[112] Han S., Shi X., Liu L., Zong L., Zhang J., Chen Q., Qian Q., Chen L., Wang Y., Jin J. (2018). Roles of B7-H3 in Cervical Cancer and Its Prognostic Value. J. Cancer.

[113] McNair C., Xu K., Mandigo A.C., Benelli M., Leiby B., Rodrigues D., Lindberg J., Gronberg H., Crespo M., De Laere B. (2018). Differential impact of RB status on E2F1 reprogramming in human cancer. J. Clin. Investig..

[114] Giacinti C., Giordano A. (2006). RB and cell cycle progression. Oncogene.

[115] Kramer K., Pandit-Taskar N., Kushner B.H., Zanzonico P., Humm J.L., Tomlinson U., Donzelli M., Wolden S.L., Haque S., Dunkel I. (2022). Phase 1 study of intraventricular (131)I-omburtamab targeting B7H3 (CD276)-expressing CNS malignancies. J. Hematol. Oncol..

[116] Y-mAbs Therapeutics 131I-Omburtamab Radioimmunotherapy for Neuroblastoma Central Nervous System/Leptomeningeal Metastases. https://clinicaltrials.gov/ct2/show/NCT03275402.

[117] MacroGenics Enoblituzumab (MGA271) in Children with B7-H3-Expressing Solid Tumors. https://clinicaltrials.gov/ct2/show/NCT02982941?term=NCT02982941&draw=2&rank=1.

[118] Memorial Sloan Kettering Cancer Center, Y-mAbs Therapeutics 131I-Omburtamab for the Treatment of Central Nervous System/Leptomeningeal Neoplasms in Children and Young Adults. https://clinicaltrials.gov/ct2/show/NCT05064306?term=NCT05064306&draw=2&rank=1.

[119] Memorial Sloan Kettering Cancer Center, Y-mAbs Therapeutics A Study of the Drug I131-Omburtamab in People with Desmoplastic Small Round Cell Tumors and Other Solid Tumors in the Peritoneum. https://clinicaltrials.gov/ct2/show/NCT04022213?term=NCT04022213&draw=2&rank=1.

[120] Nguyen P., Okeke E., Clay M., Haydar D., Justice J., O’Reilly C., Pruett-Miller S., Papizan J., Moore J., Zhou S. (2020). Route of 41BB/41BBL Costimulation Determines Effector Function of B7-H3-CAR. CD28zeta T Cells. Mol. Ther. Oncolytics.

[121] Birley K., Leboreiro-Babe C., Rota E.M., Buschhaus M., Gavriil A., Vitali A., Alonso-Ferrero M., Hopwood L., Parienti L., Ferry G. (2022). A novel anti-B7-H3 chimeric antigen receptor from a single-chain antibody library for immunotherapy of solid cancers. Mol. Ther. Oncolytics.

[122] Moghimi B., Muthugounder S., Jambon S., Tibbetts R., Hung L., Bassiri H., Hogarty M.D., Barrett D.M., Shimada H., Asgharzadeh S. (2021). Preclinical assessment of the efficacy and specificity of GD2-B7H3 SynNotch CAR-T in metastatic neuroblastoma. Nat. Commun..

[123] Tian M., Cheuk A.T., Wei J.S., Abdelmaksoud A., Chou H.C., Milewski D., Kelly M.C., Song Y.K., Dower C.M., Li N. (2022). An optimized bicistronic chimeric antigen receptor against GPC2 or CD276 overcomes heterogeneous expression in neuroblastoma. J. Clin. Investig..

[124] PersonGen BioTherapeutics (Suzhou) Co., Ltd., Tianjin Medical University Cancer Institute and Hospital, Shandong Cancer Hospital and Institute TAA06 Injection in the Treatment of Patients with B7-H3-Positive Relapsed/Refractory Neuroblastoma. https://clinicaltrials.gov/ct2/results?cond=&term=NCT05562024&cntry=&state=&city=&dist=.

[125] St. Jude Children’s Research Hospital B7-H3-Specific Chimeric Antigen Receptor Autologous T-Cell Therapy for Pediatric Patients with Solid Tumors (3CAR). https://clinicaltrials.gov/ct2/show/NCT04897321?term=NCT04897321&draw=2&rank=1.

[126] Seattle Children’s Hospital B7H3 CAR T Cell Immunotherapy for Recurrent/Refractory Solid Tumors in Children and Young Adults. https://clinicaltrials.gov/ct2/show/NCT04483778?term=NCT04483778&draw=2&rank=1.

[127] Shenzhen Geno-Immune Medical Institute, Shenzhen Children’s Hospital 4SCAR-T Therapy Targeting GD2, PSMA and CD276 for Treating Neuroblastoma. https://clinicaltrials.gov/ct2/show/NCT04637503?term=NCT04637503&draw=2&rank=1.

[128] PersonGen BioTherapeutics (Suzhou) Co., Ltd., The First Affiliated Hospital of Zhengzhou University Clinical Study of CD276 Targeted Autologous Chimeric Antigen Receptor T Cell Infusion in Patients with CD276 Positive Advanced Solid Tumor. https://clinicaltrials.gov/ct2/show/NCT04864821?term=NCT04864821&draw=2&rank=1.

[129] Shenzhen Geno-Immune Medical Institute, Sun Yat-sen University, Shenzhen Children’s Hospital Targeting CD276 (B7-H3) Positive Solid Tumors by 4SCAR-276. https://clinicaltrials.gov/ct2/show/NCT04432649?term=NCT04432649&draw=2&rank=1.

[130] Liu J.K. (2014). The history of monoclonal antibody development—Progress, remaining challenges and future innovations. Ann. Med. Surg..

[131] Pillay V., Gan H.K., Scott A.M. (2011). Antibodies in oncology. New Biotechnol..

[132] Ponziani S., Di Vittorio G., Pitari G., Cimini A.M., Ardini M., Gentile R., Iacobelli S., Sala G., Capone E., Flavell D.J. (2020). Antibody-Drug Conjugates: The New Frontier of Chemotherapy. Int. J. Mol. Sci..

[133] Mastronuzzi A., Colafati G.S., Carai A., D’Egidio M., Fabozzi F., Del Bufalo F., Villani M.F., Del Baldo G., Vennarini S., Canino C. (2022). Central Nervous System Metastasis in Neuroblastoma: From Three Decades Clinical Experience to New Considerations in the Immunotherapy Era. Cancers.

[134] Kramer K., Kushner B.H., Modak S., Pandit-Taskar N., Smith-Jones P., Zanzonico P., Humm J.L., Xu H., Wolden S.L., Souweidane M.M. (2010). Compartmental intrathecal radioimmunotherapy: Results for treatment for metastatic CNS neuroblastoma. J. Neurooncol..

[135] Ahmed M., Cheng M., Zhao Q., Goldgur Y., Cheal S.M., Guo H.F., Larson S.M., Cheung N.K. (2015). Humanized Affinity-matured Monoclonal Antibody 8H9 Has Potent Antitumor Activity and Binds to FG Loop of Tumor Antigen B7-H3. J. Biol. Chem..

[136] Kramer K., Kushner B.H., Modak S., Pandit-Taskar N., Tomlinson U., Wolden S.L., Zanzonico P., John H.L., Haque S., Souweidane M.M. (2017). A curative approach to central nervous system metastases of neuroblastoma. J. Clin. Oncol..

[137] Drago J.Z., Modi S., Chandarlapaty S. (2021). Unlocking the potential of antibody-drug conjugates for cancer therapy. Nat. Rev. Clin. Oncol..

[138] MacroGenics MGC018 versus Androgen Receptor Axis-Targeted Therapy in Participants with Metastatic Castration Resistant Prostate Cancer. https://clinicaltrials.gov/ct2/show/NCT05551117?term=NCT05551117&draw=2&rank=1.

[139] MacroGenics A Study of MGC018 in Combination with MGD019 in Participants with Advanced Solid Tumors. https://clinicaltrials.gov/ct2/show/NCT05293496?term=NCT05293496&draw=2&rank=1.

[140] Zhao L., Cao Y.J. (2019). Engineered T Cell Therapy for Cancer in the Clinic. Front. Immunol..

[141] Jayaraman J., Mellody M.P., Hou A.J., Desai R.P., Fung A.W., Pham A.H.T., Chen Y.Y., Zhao W. (2020). CAR-T design: Elements and their synergistic function. eBioMedicine.

[142] Fujiwara K., Tsunei A., Kusabuka H., Ogaki E., Tachibana M., Okada N. (2020). Hinge and Transmembrane Domains of Chimeric Antigen Receptor Regulate Receptor Expression and Signaling Threshold. Cells.

[143] Tokarew N., Ogonek J., Endres S., von Bergwelt-Baildon M., Kobold S. (2019). Teaching an old dog new tricks: Next-generation CAR T cells. Br. J. Cancer.

[144] Mehrabadi A.Z., Ranjbar R., Farzanehpour M., Shahriary A., Dorostkar R., Hamidinejad M.A., Ghaleh H.E.G. (2022). Therapeutic potential of CAR T cell in malignancies: A scoping review. Biomed. Pharmacother..

[145] Dana H., Chalbatani G.M., Jalali S.A., Mirzaei H.R., Grupp S.A., Suarez E.R., Raposo C., Webster T.J. (2021). CAR-T cells: Early successes in blood cancer and challenges in solid tumors. Acta Pharm. Sin. B.

[146] Miao L., Zhang J., Huang B., Zhang Z., Wang S., Tang F., Teng M., Li Y. (2022). Special Chimeric Antigen Receptor (CAR) Modifications of T Cells: A Review. Front. Oncol..

[147] Zhang L., Meng Y., Feng X., Han Z. (2022). CAR-NK cells for cancer immunotherapy: From bench to bedside. Biomark. Res..

[148] Pan K., Farrukh H., Chittepu V., Xu H., Pan C.X., Zhu Z. (2022). CAR race to cancer immunotherapy: From CAR T, CAR NK to CAR macrophage therapy. J. Exp. Clin. Cancer Res..

